# What immunology has to say about pesticide safety

**DOI:** 10.3389/fimmu.2024.1487805

**Published:** 2024-12-09

**Authors:** Adrián David Friedrich, Norberto Walter Zwirner

**Affiliations:** ^1^ Laboratorio de Fisiopatología de la Inmunidad Innata (IBYME-CONICET), Buenos Aires, Argentina; ^2^ Departamento de Química Biológica, Facultad de Ciencias Exactas y Naturales, Universidad de Buenos Aires, Buenos Aires, Argentina

**Keywords:** pesticides - adverse effects, immunotoxicity evaluation, cancer, anti-tumor immunity, immunosurveilance

## Abstract

The use of pesticides has enabled the development of contemporary industrial agriculture and significantly increased crop yields. However, they are also considered a source of environmental pollution and a potential hazard to human health. Despite national agencies and the scientific community analyzing pesticide safety, immunotoxicity assays are often not required, poorly designed, or underestimated. Epidemiological evidence indicates that pesticide exposure increases the risk of developing cancer. Therefore, pesticides may not only act as carcinogens *per se* but also as immunosuppressive agents that create a permissive context for tumor development. Given recent evidence demonstrating the critical role of the immune response in cancer progression, we will highlight the necessity of assessing the potential impacts of pesticides on the immune response, particularly on tumor immunosurveillance. In this Perspective article, we will focus on the need to critically review fundamental aspects of toxicological studies conducted on pesticides to provide a clearer understanding of the risks associated with exposure to these compounds to human health.

## Introduction

Plant Protection Products (PPPs) are pesticides used in food production to prevent, destroy, or control harmful organisms that could cause significant economic losses in crops. These products consist of one or more active ingredient (AI) along with excipients or co-formulants that enhance the effectiveness and stability of the AI. For a PPP to be approved, conditions must be met to ensure its safety and low toxicity for both the environment and human health. Research studies form the rational basis for the approval of PPPs in all countries. In this regard, immunotoxicity testing for pesticide evaluation has not been a priority in chemical risk assessment. In fact, the European Chemical Agency (ECHA) do not require a systematic analysis of immunotoxicity for manufactured chemicals or contaminants (1). Moreover, the US Environmental Protection Agency (EPA) published immunotoxicity testing guidelines for pesticides in 1998, but later stated that immunotoxicity requirements should be simplified (2). Immunotoxicity studies conducted by the EPA are designed to assess the immunosuppressive potential of chemicals by measuring antibody production in response to sheep red blood cells in mice or rats. Additionally, standard subchronic and chronic toxicology studies typically provide data on organ weights, pathological and histopathological examinations of immune system organs and tissues, and differential white blood cell counts. In some cases, serum immunoglobulin levels may also be included. However, as the EPA itself stated “there is concern that these endpoints alone may be insufficient to fully characterize the potential for immunotoxicity, as they do not directly evaluate the functional capacity of immune components” (2). Even though each country is responsible for the safety of PPPs used in each territory, there is no doubt that both the EPA and the European Food Safety Authority (EFSA) in the European Union have a strong influence on national regulatory agencies worldwide.

In this Perspective article, we will focus on the need to critically review fundamental aspects of toxicological studies conducted on PPPs to provide a clearer understanding of the risks associated with exposure to these compounds on human health. We will argue that there is sufficient evidence to suggest that humans highly exposed to PPPs, like conventional farmers or people living near to fumigated fields, are more susceptible to cancer development. Given the evidence from recent years demonstrating the critical role of the immune response in cancer protection, we will highlight the necessity of assessing the potential impacts of PPPs on the immune response, particularly on tumor immunosurveillance. Sustained by bibliography and unpublished data from our lab that reveals how PPPs negatively impact on the immune system, we will state that current strategies to evaluate the carcinogenic potential of PPPs are insufficient.

## The carcinogenic potential of PPPs: current evaluation strategies and their limitations

The carcinogenic potential of a PPPs is currently evaluated using animal models exposed to AIs. After prolonged treatment, the presence of neotransformed cells in different organs and tissues is assessed through histopathological analysis. In the following sections, we will present several reasons why this strategy is insufficient and requires significant changes to accurately assess human health risks.

## PPPs are much more than just active ingredients

As mentioned, PPPs are commercial formulations that contain not only an AI but also co-formulants. Since co-formulants are considered inert, companies are not required to disclose which excipients they include in their formulations. Consequently, the toxicity evaluation of pesticides is typically performed by exposing experimental animals only to pure AI. However, there is concerning evidence suggesting this approach is inappropriate. *In vitro* studies using human cell lines have demonstrated that glyphosate-based herbicides (GbH) are far more toxic than glyphosate (GLY) alone, even at environmentally relevant doses (3). This increased toxicity is likely due to some co-formulants acting as surfactants, which potentiate the permeability and entry of GLY into target cells. Moreover, certain co-formulants present in GbH, such as polyoxyethylene tallow amine (POEA) surfactants, have been shown to be more toxic than GLY itself in amphibians (4), *Drosophila* (5) and human cell lines (6). In 2016, the EU Commission recommended to Member States that POEA-type co-formulants be banned from use in GBHs (7). Despite this alarm signal, the toxic effects of PPPs excipients remain understudied and unevaluated, and companies are still not forced to disclose excipient’s identity.

In conclusion, the toxicological effects should be widened to include the evaluation of their carcinogenicity and performed using complete commercial formulations that include both AIs and co-formulants.

## Epidemiology studies in highly fumigated countries are scarce

The use of pesticides has enabled the development of contemporary industrial agriculture (8) and has significantly increased crop yields. However, they are considered a source of environmental pollution and a potential hazard to human health. According to official FAO data, the per capita use of agricultural pesticides in Argentina has increased 5.7 times from 1990 to 2020 (https://www.fao.org/faostat/es/#compare). Since Argentina’s food production is massively exported to Europe and Asia, the presence of pesticides in food could have public health implications for populations beyond Argentina’s borders. Besides, 83% of European soils contained at least 1 pesticide residue and 166 different pesticide mixtures were detected (9). The SPRINT project (https://sprint-h2020.eu/), funded by the EU, was launched in 2020 with the aim of “developing a Global Health Risk Assessment Toolbox to evaluate the impacts of Plant Protection Products (PPPs) on the environment and human health, and proposing several transition pathways.” Since its inception, several scientific articles have been published as part of the SPRINT project, examining the presence and levels of pesticides in the environment, animals, and human samples across Europe and Argentina. Notably, a median of 20 pesticides were detected in water samples from various European countries, 38% of which are banned in the EU. Similar findings were reported in samples from Argentina. The situation varies significantly between countries. Croatia showed the lowest pesticide levels, with 67% and 33% of water samples containing 10–20 and fewer than 10 PPPs, respectively. At the opposite end, France exhibited higher contamination levels, with 83% and 17% of samples containing 20–30 and 31–40 different pesticides, respectively. While the SPRINT project is highly valuable to be aware of pesticide exposure levels in Europe and Argentina, it does not evaluate the effects of PPPs on human health. Moreover, Dr. Maria Valeria Ame’s group has been studying environment pollutants and assessing ecological risks in Argentina for over a decade. In a recent publication, they identified insecticides, herbicides, and fungicides as the primary contributors to ecological risk in water from three rivers in Córdoba province. Notably, in river sediments, they observed a very high risk in the lower basin, primarily driven by the contribution of aminomethylphosphonic acid (AMPA), the primary metabolite of glyphosate (GLY) (10).

In this regard, several studies have associated occupational pesticide exposure in farmers with various types of cancer, such as breast cancer (11), acute myeloid leukemia (12) and non-Hodgkin lymphoma (13). Additionally, there are articles demonstrating an increased cancer risk in towns or regions where large quantities of pesticides are used (14, 15). In 2017, researchers from Argentina analyzed pesticide levels in Monte Maíz, Córdoba, a town surrounded by fumigated fields (16). They found not only high levels of pesticide mixtures in crop field soil but also in children’s playground soil collected from places located well within the town. Alarmingly, the authors showed that cancer occurs earlier in life, is more frequent, and is more deadly in Monte Maíz than in Córdoba city, an urban, highly populated city located 290 km away from Monte Maíz.

Nontheless, studies analyzing cancer biomarkers in exposed individuals, where specific pesticides are measured in their biological fluids, are lacking. Such studies are essential to precisely and timely understand the extent and depth of the problem.

A study conducted in 2021 used a multiethnic cohort to investigate the association between urine concentrations of AMPA and breast cancer (17). The authors found a 4.5-fold higher risk of developing breast cancer in individuals in the highest versus the lowest quintile of AMPA excretion.

To our knowledge, the are no published studies addressing the potential association of pesticide residues in human biological fluids with immune disfunction that might explain the higher cancer risk observed in highly exposed individuals.

## Studies analyzing a single PPP are insufficient

Results published by participating groups in the SPRINT project have demonstrated that, among the 20 most frequently detected pesticides in human blood samples, three are classified as carcinogens, seven as endocrine disruptors, and six have negative effects on reproduction and development. In this article, they state that “efforts are needed to elucidate the unknown effects of mixtures” (18). Moreover, in a study conducted in 2023 (19), the frequency of detection of pesticide mixtures in the urine of farmers from five European countries was determined. In 84% of the analyzed samples at least two different pesticides were detected, the median number of detected pesticides in the urine samples was 3, with a maximum of 13 pesticides detected in a single sample. Based on these results, it is now clear that an approach based on assessing carcinogenicity of a single PPP is no longer representative of environmental exposure. However, experimental studies focusing on single PPP are necessary to fully elucidate toxicity mechanisms exerted by particular pollutants.

## The potential erosion of the immune response by PPPs is understudied

Pesticides are known to exert immunotoxic effects *in vitro* (20), yet evidence of such effects on humans exposed to long-term, environmentally relevant doses is limited. *In vitro* studies have shown that pesticides, including atrazine, organophosphate (OP) compounds, carbamates, and pyrethroids, disrupt the function of B and T lymphocytes, as well as natural killer (NK) cells and macrophages. Few studies have investigated the immunotoxicity of pesticides using *in vivo* models. In one case, animals were exposed to the insecticide chlorpyrifos (CPF) at 1/5, 1/10, and 1/20 of its LD50 value for 45 days. During this period, CPF reduced T cell frequency in a dose-dependent manner (21). Moreover, CPF-treated mice exhibited an increased frequency of NK and B cells, although functional and phenotypic parameters were not assessed. Additionally, it remains unclear whether this treatment affected the immune response capacity to deal with challenges as pathogens or tumor cells. It is worth noting that experimental animal models should rigorously reflect real-life human environmental exposure. This includes not only environmentally relevant doses but also the most likely routes of entry, exposure durations, different life stages during exposure, and the effects of pesticide mixtures, among other factors.

Pesticides, primarily atrazine but also other PPP, have been shown to act as endocrine-disrupting chemicals (EDCs), meaning they negatively impact the endocrine system of non-target organisms, including humans. Once in the body, EDCs compete with endogenous hormones through various receptors and affect a pethora of cellular pathways.

For decades, it has been evident that the endocrine and immune systems are intricately connected, suggesting that PPP with EDC properties may indirectly affect the immune system by altering hormone levels. Atrazine, the second most widely used herbicide globally, stimulates the hypothalamic-pituitary-adrenal axis, ultimately leading to increased levels of immunosuppressive glucocorticoids (22). While atrazine does not directly interact with estrogen receptors, it enhances aromatase activity (23) resulting in elevated serum estrogen levels in male rats (24). High levels of estrogen are known to alter T cell function, promoting a Th2 differentiation (25), and reduce NK cytotoxicity (26). In addition to the indirect effects of endocrine disruption on the immune system, many studies have demonstrated direct effects of PPP on immune cells via endocrine-related receptors. For example, immature immune cells in the thymus express G-protein coupled estrogen receptor 1 (GPER-1) (27) which can sense atrazine (28), suggesting that normal immune cell development may be sensitive to atrazine exposure. Furthermore, Brusing et al. demonstrated that GPER-1 stimulation in mature CD4+ T cells induces IL-10 production *in vitro* (29), linking GPER-1 agonism to immunoregulatory effects.

In 2002 the cancer immunoediting theory was first introduced, highlighting the crucial role of the immune system in cancer progression (30). Schreiber and colleagues proposed a model wherein tumor establishment occurs through three sequential phases: the elimination phase, the equilibrium phase, and the escape phase. Initially, during the elimination phase, NK cells and antigen-specific CD4^+^ and CD8^+^ T cells eradicate tumor cells. If cancer cells are not completely eliminated, the surviving cancer cells establish an equilibrium with the immune system, characterized by a balance between the tumor elimination functions of immune cells and the ability of tumor cells to remain overlooked by the immune system. Later (perhaps after several months or years), tumor cells coopt several immunoregulatory mechanisms to promote immune escape, resulting in an uncontrolled tumor growth leading to clinically detectable tumors. During these events, tumor cells that survive and proliferate in the presence of anti-tumor immune cells undergo a selection process known as immunoediting.

There is a growing concern that pesticides may act not only as carcinogens but also exert detrimental effects on the immune system, creating a permissive environment for nascent tumor cells. Despite these abundant epidemiological data, studies exploring the impact of pesticides on anti-tumor immune cell functions are limited.

In an article published in Spain this year, the authors compared serum cytokines as biomarkers of immune function from 111 greenhouse farmers occupationally exposed to pesticides during high and a low pesticide exposure period with 79 non-exposed individuals (31). Interestingly, they found that the farmers group had higher levels of Th2 cytokines (such as IL-4, IL-6, IL-13 and RANTES) than the non-exposed group. Notably, the concentration of those immune mediators was higher during the high exposure period in both groups. It is worth noting that when the balance of the immune response is shifted to a Th2 profile in people living in developed countries, it manifests as asthma and allergy (32). Moreover, Th2-associated cytokines, such as IL-6, have been associated with inefficient immune responses to tumors worsening the prognosis in hepatocellular carcinoma (33), pancreatic cancer (34) and ovarian cancer patients (35). Th2-associated cytokine can shape tumor microenvironment by several mechanisms such as the induction of the differentiation of macrophages to an anti-inflammatory (M2) profile (36) and indirectly enhance the metastatic capacity of cancer cells (37).

A similar study was conducted in 2020 in Brazil (38), but assessing different immune populations and cytokines in the blood of farmers and control individuals. As the Spanish article, Brazilian farmers exposed to pesticides had increased concentrations of IL-6. Strikingly, a significant increase in the number of classical monocyte and dendritic cells in the exposed group was observed as well as in total T cells, central memory CD8^+^ T cells and effector memory CD8^+^ T cells. These results reinforce the fact that pesticide exposure skews the immune response towards a profile with reduced efficacy to control nascent tumor cells and cancer progression. However, we ignore if and how these effects may impact on cancer development.

In a recent publication, cytokine and OP metabolite levels were measured in the blood of flower workers from Mexico. In contrast to Spanish and Brazilian farmers, Mexican farmers with higher serum OP metabolite levels showed a decrease in pro-inflammatory cytokines such as IL-6, IFN-γ, and TNF and an increase in IL-10 compared to farmers with low or non-detectable OP metabolites (39).

Unpublished data from our lab indicate that Roundup^®^ (Bayer), a worldwide-used GbH, when combined with Clorpi48 (Huagro) a chlorpyrifos-based insecticides (CbI), adversely impact on human NK cells from healthy donors. Environmentally relevant exposure doses of these pesticides resulted in a reduced capacity of cytokine-stimulated NK cells to secrete interferon-gamma (IFN-γ). Furthermore, the combination of GbH and CbI impaired the cytotoxic capacity of NK cells against the tumor cell line K562. Regarding the adaptive immune response, our results indicate that the combination of GbH and CbI negatively affected T cell proliferation and differentiation *in vitro* to Th1 cells.

Altogether, data about the detrimental effects of pesticide mixtures on immune cell functions critical for immunosurveillance are growing and may help explain the increased cancer incidence observed in populations residing in areas exposed to pesticide fumigation.

## Conclusions and future perspectives

Considering the epidemiological evidence of higher cancer incidence in individuals exposed to PPPs and the recent discoveries in basic immunology, it is clear that current toxicity testing of PPPs is insufficient and needs reassessment. Commercial pesticide formulations and pesticide mixtures should be evaluated through functional assays that monitor anti-tumoral effector functions using both *in vitro* and *in vivo* approaches. Moreover, epidemiological studies analyzing cancer biomarkers and immune cell functions in individuals exposed to environmental pesticides would be extremely valuable. Notably, the ENDOMIX project (https://endomix.eu), launched in January 2024, aims to evaluate the immunotoxicity of endocrine-disrupting chemicals (EDCs) found in the environment across seven European countries. This project has received a grant of over 6 million euros highlighting the significant concern surrounding the impact of environmental pollutants on the immune system. Many plant protection products (PPPs), such as glyphosate-based herbicides (GbHs), organophosphate pesticides (OPs), carbamates, and neonicotinoids, have been shown to act as EDCs (40–43). It is therefore expected that the ENDOMIX project will consider PPPs as relevant environmental chemicals capable of affecting the immune system. While this type of study is considerably more resource-intensive and expensive than traditional epidemiological approaches, it offers invaluable insights, as discussed in this article. If toxicity evaluations of PPPs do not change to include immunotoxicity functional assays, it is likely that mid- and/or long-term effects may arise in exposed individuals and impact on the incidence of different types of cancer in exposed communities.

## Data Availability

The results constitute unpublished data and will be send for publication in the near future. Requests to access the datasets should be directed to Adrian Friedrich, afriedrich.bioq@gmail.com.
